# Serotonin Modulates Stellate Cell Excitability via 5-HT Receptors and HCN Channels in the Mouse Anteroventral Cochlear Nucleus

**DOI:** 10.3390/ijms27073030

**Published:** 2026-03-26

**Authors:** Beytullah Özkaya, Caner Yıldırım, Ender Erdoğan, Mehmet Şerif Aydın, Ramazan Bal

**Affiliations:** 1 Faculty of Medicine, University of Adıyaman, Adıyaman 02040, Türkiye; bozkaya@adiyaman.edu.tr; 2 Faculty of Medicine, University of Gaziantep, Gaziantep 27310, Türkiye; cyildirim@gantep.edu.tr; 3 Faculty of Medicine, Selçuk University, Konya 42080, Türkiye; drender@selcuk.edu.tr; 4 Faculty of Medicine, İstanbul Medipol University, İstanbul 34810, Türkiye; msaydin@medipol.edu.tr

**Keywords:** cochlear nucleus, patch clamp, 5-HT receptors, stellate cells, HCN, brain slices

## Abstract

Serotonergic projections innervate both the dorsal and ventral cochlear nuclei; however, the electrophysiological consequences of serotonergic input in the ventral cochlear nucleus (VCN) remain incompletely understood. This study aimed to identify the serotonin receptor subtypes involved in serotonergic modulation of stellate cells in the mouse anteroventral cochlear nucleus (AVCN) and to determine the underlying ion channel mechanisms. Whole-cell patch-clamp recordings were performed in acute brain slices obtained from postnatal day 12–17 mice. Bath application of serotonin (25 µM) induced membrane depolarization (~5 mV) and increased action potential firing. Pharmacological experiments demonstrated that antagonists of 5-HT1A, 5-HT2A, and 5-HT2C receptors partially reversed the depolarization and reduced serotonin-induced inward currents, indicating that multiple receptor subtypes contribute to serotonergic excitation. Blockade of hyperpolarization-activated cyclic nucleotide-gated (HCN) channels with extracellular Cs^+^ suppressed approximately 95% of the serotonin-induced depolarization and inward current, implicating HCN channel-mediated Ih as a principal ionic mechanism. Serotonin significantly increased Ih amplitude. Analysis of steady-state activation revealed no statistically significant shift in V0.5; however, under near-resting membrane potential conditions, serotonin significantly reduced the slope factor of the activation curve, consistent with altered voltage sensitivity of Ih gating. Immunohistochemical analysis confirmed the presence of 5-HT1A, 5-HT2A, and 5-HT2C receptors in the AVCN. Together, these findings indicate that serotonergic excitation of AVCN stellate cells is mediated by coordinated activation of multiple 5-HT receptor subtypes and primarily involves modulation of HCN-dependent subthreshold membrane dynamics.

## 1. Introduction

The cochlear nucleus (CN) is located at the border between the pons and the medulla oblongata at the entrance of the auditory nerve root and is responsible for processing and relaying auditory information to the higher-order auditory nuclei [1]. CN integrates and processes acoustic information coming from the cochlea via separate, well-defined populations of neurons, which process basic qualities of sound, such as intensity, frequency, and temporal coding and transmit to the upper auditory nuclei [2]. Anatomically, the cochlear nucleus consists of two parts, the ventral cochlear nucleus (VCN) and the dorsal cochlear nucleus (DCN), and each part contains different cell types [3]. In studies conducted on sections obtained from the ventral cochlear nucleus tissue, three cell types with different biophysical and morphological properties have been characterized [4,5]. Of these cells, the octopus cells are located in the posteroventral cochlear nucleus (PVCN), while the stellate and bushy cells are located especially in the anteroventral part of the cochlear nucleus (AVCN) [2,6,7]. Stellate and bushy cells are intermingled and have contrasting responses to depolarizing current pulses [8,9]. Stellate cells fire regular trains of action potentials with equal interspike intervals (chopper firing pattern) in response to depolarizing currents. On the other hand, bushy cells fire one or two action potentials at the onset of depolarizing square current pulses [8,9].

In the central nervous system, most axonal projections of serotonergic neurons originate from the dorsal and median raphe nuclei, and adjacent nuclei in the lower brainstem and terminate in the cortical, limbic, midbrain, and hindbrain regions [10]. The serotonergic system modulates diverse physiological and behavioral functions, such as sleep, feeding, nociception, mood and emotion [11]. It is known that the serotonergic system also affects auditory brain regions. It has been shown that auditory signals are modulated by the serotonergic system in the auditory cortex, inferior colliculus, lateral superior olivary nucleus, medial nucleus of the medial trapezoid body, superior olivary complex, ventral nucleus of the trapezoid body and medial geniculate body neurons [12,13,14,15,16]. There is evidence that disturbance of the serotonergic system contributes to disorders related to the auditory system [17,18,19]. Although tinnitus is generally associated with excessive spontaneous excitability of fusiform cells from the DCN neurons, it has been suggested that excessive excitability of the AVCN neurons may also contribute to tinnitus [20,21,22]. Although serotonin increases neuronal excitability in fusiform neurons in the DCN, it has been shown to inhibit neuronal activity in other auditory nuclei [16]. Studies have shown the presence of serotonergic fibers in the dorsal and ventral cochlear nuclei [12,23]. However, the electrophysiological effect of serotonergic innervation in the VCN remains incompletely understood. Tinnitus is the perception of sound in the absence of an external acoustic stimulus and is commonly associated with altered neuronal excitability and abnormal spontaneous activity within the auditory pathway [24]. It has been suggested that hyperexcitability of AVCN neurons may contribute to tinnitus [20,21,22]. Therefore, understanding the normal physiological effects of 5-HT in the auditory system may provide insight into normal brain function and suggest new approaches in the treatment of tinnitus.

Hyperpolarization-activated cyclic nucleotide-gated (HCN) ion channels play a crucial role in regulating the membrane excitability of cardiac and neuronal cells [25,26]. The currents produced by HCN channels have been known as Ih (or I(f) or I(q)) [26]. In the DCN principal cells, the ion channel mediating the excitatory effect caused by serotonin has been identified as HCN [27]. In addition, the function of HCN channels in the excitability of PVCN octopus cells and in the primary auditory neurons of the mouse inner ear has been revealed [28,29]. The role of HCN channels in serotonergic modulation of AVCN stellate cells has not been studied. Therefore, the aim of this study was to identify the serotonin receptor subtypes mediating serotonergic effects and to determine the ion channel mechanisms underlying serotonin-dependent modulation of stellate cell excitability in the AVCN.

## 2. Results

### 2.1. Effect of Serotonin on Stellate Cells

**Figure 1:**
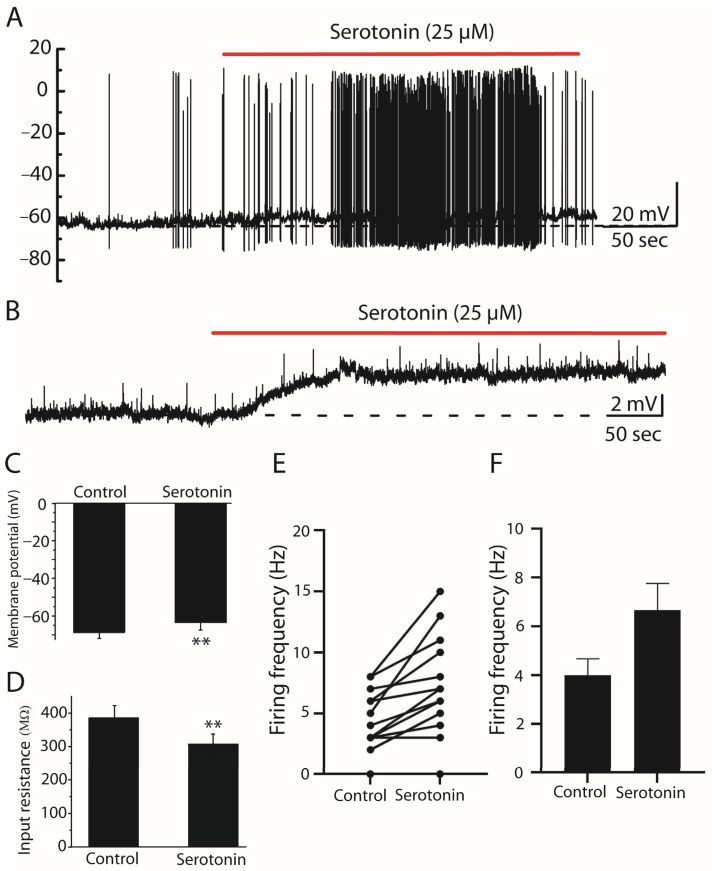
Effect of serotonin (25 µM) on spontaneous activity of stellate cells in current-clamp recordings. (**A**) Representative recording showing that bath application of serotonin caused a depolarization of the resting membrane potential by approximately 5 mV and increased spontaneous firing activity in stellate cells. (**B**) Serotonin was applied in the presence of synaptic blockers (DNQX, APV, and strychnine) and the sodium channel blocker tetrodotoxin (TTX), producing a comparable depolarization. (**C**) Quantification of membrane potential changes showing that serotonin significantly depolarized stellate cells. (**D**) Summary data showing the effect of serotonin on input resistance. Serotonin significantly decreased input resistance compared with control conditions. (**E**) Paired plot showing the increase in firing frequency in individual cells following serotonin application. Each dot represents an individual cell; lines connect paired recordings obtained from the same neuron. (**F**) Summary bar graph illustrating the increase in mean firing frequency after serotonin application. Data are presented as mean ± SEM. ** *p* < 0.01.

In current-clamp recordings, serotonin (25 µM) was bath-applied in the perfusion solution (aCSF) to examine its effects on stellate cells. Resting membrane potential, input resistance, membrane capacitance, membrane time constant, and firing activity were compared before and after serotonin application. Bath application of serotonin produced a significant depolarization of the resting membrane potential by approximately 4.93 mV, shifting from −68.26 ± 0.95 mV under control conditions to −63.32 ± 1.09 mV following serotonin application (*n* = 15, paired Student’s *t*-test, *p* < 0.001, Figure 1A,C). Since serotonin activates receptors on the cell membrane and increases membrane conductance by opening ion channels, it also significantly decreased the input resistance of stellate cells from 385.14 ± 37.69 MΩ to 303.79 ± 33.39 MΩ (*n* = 14, paired Student’s *t*-test, *p* < 0.001) (Figure 1D). Input resistance was estimated from the steady-state voltage deflection produced by hyperpolarizing current injections using Rin = ΔV/ΔI (see Methods). The serotonin-induced depolarization was accompanied by an increase in spontaneous firing activity (Figure 1A). Quantitative analysis showed that the mean firing frequency increased from 4.00 ± 0.67 Hz under control conditions to 6.67 ± 1.11 Hz after serotonin application; however, this increase did not reach statistical significance (*n* = 15, paired Student’s *t*-test, *p* > 0.05, Figure 1E,F). To determine whether the excitatory effects of serotonin were mediated directly through postsynaptic receptors or indirectly via presynaptic network activity, serotonin (25 µM) was applied in the presence of the sodium channel blocker tetrodotoxin (TTX) together with synaptic blockers (DNQX, APV, and strychnine). Under these conditions, serotonin still induced a comparable depolarization, indicating that its excitatory action on stellate cells is mediated predominantly through postsynaptic mechanisms (Figure 1B). Together, these findings indicate that serotonin depolarizes stellate cells and increases their excitability through direct postsynaptic mechanisms.

**Figure 2:**
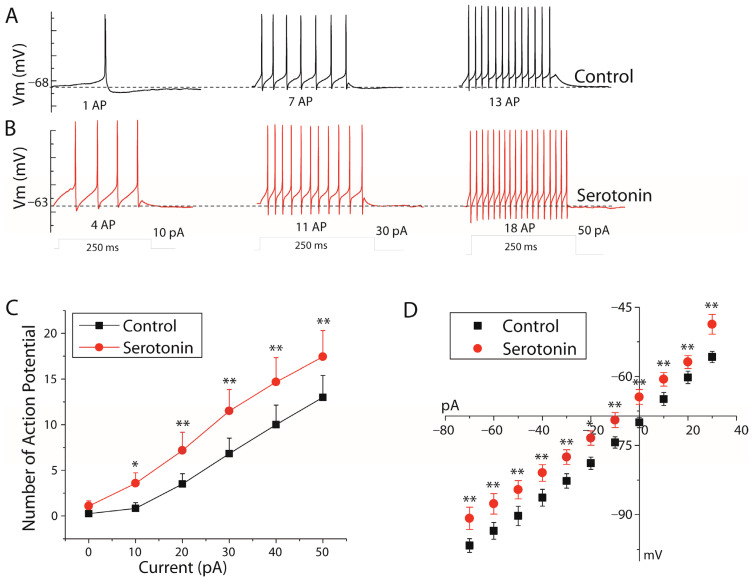
Effect of serotonin (25 µM) on the number of action potentials and current–voltage relationship in the stellate cell. (**A**) Number of action potentials in response to DC current stimuli before serotonin application. (**B**) Number of action potentials after serotonin application. (**C**) Linear relationship between injected currents and action potential number (*n* = 11). (**D**) Current–voltage relationship before and after serotonin application (*n* = 11, paired Student’s *t*-test, * *p* < 0.05; ** *p* < 0.001).

Serotonin significantly altered spike responses to step current injections in stellate cells. Bath application of serotonin (25 µM) depolarized stellate cells and increased the number of action potentials in each depolarizing step (*n* = 15, paired Student’s *t*-test, *p* < 0.001, Figure 2A,B). The number of action potentials at each current level and the membrane potential as a function of injected current are shown in Figure 2C and Figure 2D, respectively.

Although serotonin induced spontaneous firing in some stellate cells (as illustrated in Figure 1A), this effect was not observed uniformly across all recorded neurons. At 0 pA, serotonin increased spontaneous firing from 0.25 ± 0.25 to 1.08 ± 0.57 action potentials (*n* = 11), although this increase did not reach statistical significance (*n* = 11, paired Student’s *t*-test, *p* > 0.05), likely due to intercellular variability.

Although serotonin reduced input resistance, it simultaneously depolarized the resting membrane potential and increased subthreshold excitability. Therefore, the net effect of serotonin on action potential output reflects the combined influence of (i) a depolarizing shift toward spike threshold and (ii) modulation of voltage-dependent conductances, rather than input resistance alone.

### 2.2. Contribution of 5-HT1A, 5-HT2A, and 5-HT2C Receptors to Serotonin-Induced Excitation

The serotonin receptor subtypes mediating these excitatory effects were pharmacologically identified using specific antagonists in current- and voltage-clamp experiments. Previous studies have shown that 5-HT1A, 5-HT2A, and 5-HT2C receptors are expressed in the AVCN [30,31,32]. However, their role in modulating the electrophysiological properties of stellate cells had not been determined.

**Figure 3:**
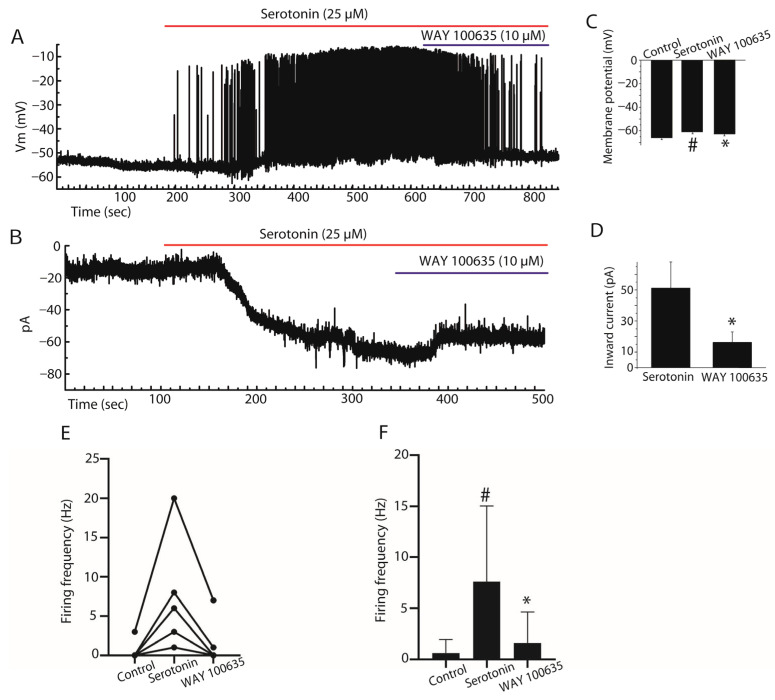
Contribution of 5-HT1A receptors to serotonin-induced excitation of stellate cells. (**A**) Under current clamp, serotonin depolarized the membrane and increased spontaneous firing. WAY 100635 partially blocked the depolarization (~2 mV) and reduced firing frequency. (**B**) Under voltage clamp, serotonin induced an inward current of ~50 pA at holding potential of −70 mV, close to resting membrane potential, which was reduced by WAY 100635 (~16 pA). (**C**) Summary bar graph showing membrane potential changes under control conditions, after serotonin application, and after addition of WAY 100635. (**D**) Quantification of serotonin-induced inward currents and their reduction by WAY 100635. (**E**) Paired dot plot showing changes in firing frequency in individual stellate cells under the same conditions. (**F**) Summary bar graph illustrating mean firing frequency values in control, serotonin and WAY 100635 conditions. Data are presented as mean ± SEM. * *p* < 0.05. # *p* < 0.05 vs. control; * *p* < 0.05 vs. serotonin.

Bath application of serotonin (25 µM) induced a depolarization of 5.66 ± 0.48 mV in stellate cells under current clamp. In the presence of serotonin (25 µM), application of the 5-HT1AR antagonist WAY 100635 (10 µM) reversed this depolarization by 2.02 ± 0.58 mV and led to a decrease in their spontaneous firing frequency (*n* = 5, paired Student’s *t*-test, *p* < 0.001, Figure 3A,C,E,F). Similarly, under voltage clamp, serotonin induced an inward current of 51.25 ± 33.68 pA, and WAY 100635 blocked 16.25 ± 12.68 pA of this current (*n* = 4, paired Student’s *t*-test, *p* < 0.05, Figure 3B,D).

Consistent with these findings, quantitative analysis of firing frequency revealed that serotonin markedly increased firing from 0.6 ± 0.6 Hz under control conditions to 7.6 ± 3.32 Hz, whereas application of WAY-100635 reduced firing frequency to 1.6 ± 1.36 Hz (*n* = 5, paired Student’s *t*-test, *p* < 0.05, Figure 3E,F). Together, these results indicate that 5-HT1A receptors contribute to the depolarizing and excitatory effects of serotonin on stellate cells.

**Figure 4:**
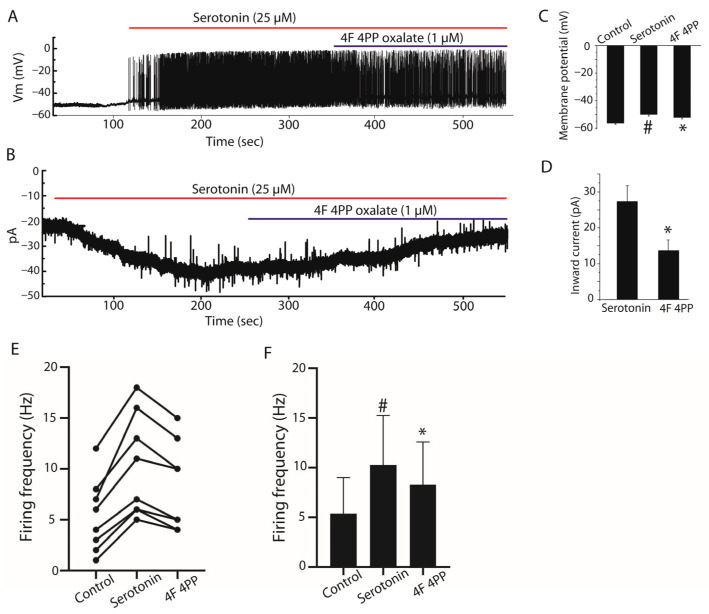
Contribution of 5-HT2A receptors to serotonin-induced responses in stellate cells. (**A**) Representative current-clamp recordings showing membrane responses of stellate cells under control conditions, after bath application of serotonin (25 µM), and in the presence of the selective 5-HT2A receptor antagonist 4F 4PP oxalate (1 µM). Serotonin depolarized the membrane potential and increased firing activity, whereas 4F 4PP oxalate partially reversed these effects. (**B**) Representative voltage-clamp recordings showing the inward current induced by serotonin and its partial inhibition by 4F 4PP oxalate. (**C**) Summary bar graph showing membrane potential changes under control conditions, after serotonin application, and in the presence of 4F 4PP oxalate (*n* = 8). (**D**) Quantification of serotonin-induced inward currents and their reduction by 4F 4PP oxalate (*n* = 10). (**E**) Paired dot plot showing changes in firing frequency in individual stellate cells under the same conditions. (**F**) Summary bar graph illustrating mean firing frequency values in control, serotonin, and 4F 4PP oxalate conditions (*n* = 8). Data are presented as mean ± SEM. # *p* < 0.05 vs. control; * *p* < 0.05 vs. serotonin.

The involvement of 5-HT2A receptor in the serotonin-induced depolarization in current clamp and the current in voltage clamp, the specific antagonist of 5-HT2A receptor, 4F 4PP oxalate (1 µM) was tested. Under current clamp, serotonin induced a depolarization of 6.15 ± 1.84 mV, and 4F 4PP oxalate blocked 2.07 ± 0.99 mV of this depolarization (*n* = 8, paired Student’s *t*-test, *p* < 0.001, Figure 4A,C). Under voltage clamp, serotonin induced an inward current of 27.32 ± 14.22 pA, and 4F 4PP oxalate blocked ~13.64 pA of this current (*n* = 10, paired Student’s *t*-test, *p* < 0.001, Figure 4B,D). Consistent with the membrane depolarization, serotonin significantly increased the firing frequency of stellate cells from 5.38 ± 1.28 Hz under control conditions to 10.25 ± 1.77 Hz. In the presence of the selective 5-HT2A receptor antagonist 4F 4PP oxalate, firing frequency was reduced to 8.25 ± 1.53 Hz, indicating a partial attenuation of the serotonergic effect (*n* = 8, paired Student’s *t*-test, *p* < 0.05, Figure 4E,F).

**Figure 5:**
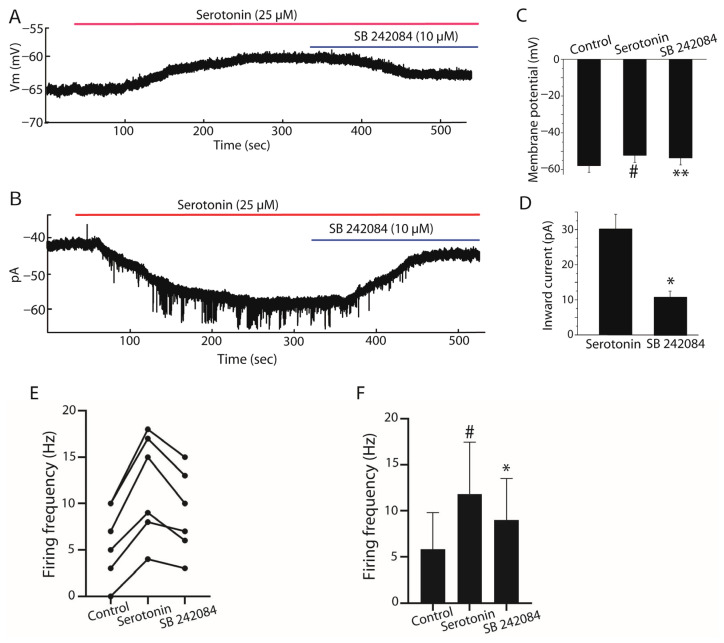
Contribution of 5-HT2C receptors to serotonin-induced responses in stellate cells. (**A**) Representative current-clamp recordings showing membrane responses of stellate cells under control conditions, after bath application of serotonin (25 µM), and in the presence of the selective 5-HT2C receptor antagonist SB 242084. Serotonin depolarized the membrane potential, and SB 242084 partially reversed this depolarization. In this representative recording, spontaneous firing was not observed under the specific recording conditions. (**B**) Representative voltage-clamp recording showing the inward current induced by serotonin and its partial inhibition by SB 242084. (**C**) Summary bar graph showing membrane potential changes under control conditions, after serotonin application, and in the presence of SB 242084 (*n* = 6). (**D**) Quantification of serotonin-induced inward currents and their reduction by SB 242084 (*n* = 7). (**E**) Paired dot plot showing changes in firing frequency in individual stellate cells under the same conditions. (**F**) Summary bar graph illustrating mean firing frequency values in control, serotonin, and serotonin + SB 242084 conditions (*n* = 6). Data are presented as mean ± SEM. # *p* < 0.05 vs. control; ** *p* < 0.001, * *p* < 0.05 vs. serotonin.

To reveal the effect of 5-HT2C receptor on serotonin-induced excitatory effect in stellate cells, SB 242084 (specific antagonist of 5-HT2C receptor) was tested. Serotonin depolarized stellate cells by 5.65 ± 0.35 mV, and SB 242084 blocked 2.5 ± 0.6 mV of this depolarization (*n* = 6, paired Student’s *t*-test, *p* < 0.001, Figure 5A,C). In this representative recording, spontaneous firing was not observed under these specific recording conditions, reflecting variability among stellate cells. Under voltage clamp at holding potentials of −70 mV, serotonin induced an inward current of 30.14 ± 11.1 pA, and SB 242084 blocked 10.86 ± 4.1 pA of this current (*n* = 7, paired Student’s *t*-test, *p* < 0.001, Figure 5B,D). Consistent with these findings, serotonin increased firing frequency from 5.83 ± 1.62 Hz under control conditions to 11.83 ± 5.64 Hz, whereas application of SB 242084 reduced firing frequency to 9.00 ± 1.84 Hz (*n* = 6, paired Student’s *t*-test, *p* < 0.05, Figure 5E,F).

### 2.3. Serotonin Modulates Ih Current in Stellate Cells

**Figure 6:**
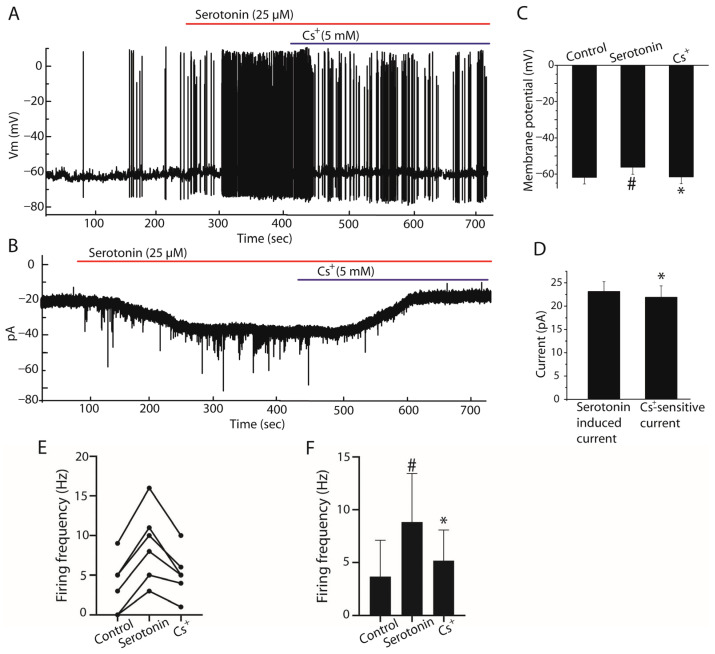
Involvement of HCN channel-mediated Ih currents in serotonin-induced excitation of stellate cells. (**A**) Representative current-clamp recordings showing membrane responses of stellate cells under control conditions, after bath application of serotonin (25 µM), and following application of extracellular Cs^+^ (5 mM). Serotonin depolarized the membrane potential and increased spontaneous firing activity, whereas Cs^+^ partially reversed these effects. (**B**) Representative voltage-clamp recordings showing the inward current induced by serotonin and its reduction by Cs^+^. (**C**) Summary bar graph showing membrane potential changes under control conditions, after serotonin application, and in the presence of Cs^+^ (*n* = 6). (**D**) Quantification of serotonin-induced inward current and the Cs^+^-sensitive component of this current (*n* = 6). (**E**) Paired dot plot showing changes in firing frequency in individual stellate cells under the same conditions. (**F**) Summary bar graph illustrating mean firing frequency values in control, serotonin and Cs^+^ conditions (*n* = 6). Data are presented as mean ± SEM. # *p* < 0.05 vs. control; * *p* < 0.05 vs. serotonin.

After determining the effects of serotonin on AVCN stellate cells and identifying the receptor subtypes mediating this response, we next investigated the ionic mechanism underlying the serotonergic excitation. Under current-clamp conditions, serotonin depolarized stellate cells by 5.64 ± 0.15 mV. In the presence of extracellular Cs^+^ (5 mM), a known blocker of HCN channel-mediated Ih currents, 5.37 ± 0.15 mV of this depolarization was suppressed (*n* = 6, paired Student’s *t*-test, *p* < 0.05, Figure 6A,C). Similarly, under voltage-clamp conditions at a holding potential of −70 mV, serotonin induced an inward current of 23.09 ± 2.07 pA, of which 21.93 ± 2.19 pA was Cs^+^-sensitive (*n* = 6, paired Student’s *t*-test, *p* < 0.05, Figure 6B,D). Consistent with these findings, serotonin significantly increased firing frequency compared with control conditions, whereas application of Cs^+^ reduced this increase (*n* = 6, paired Student’s *t*-test, *p* < 0.05, Figure 6E,F). Together, these results indicate that the serotonergic inward current is largely mediated by Cs^+^-sensitive conductances, suggesting that HCN channel-mediated Ih currents contribute substantially to the excitatory effects of serotonin in stellate cells.

### 2.4. Serotonin Enhances Voltage Sag in Stellate Cells

**Figure 7:**
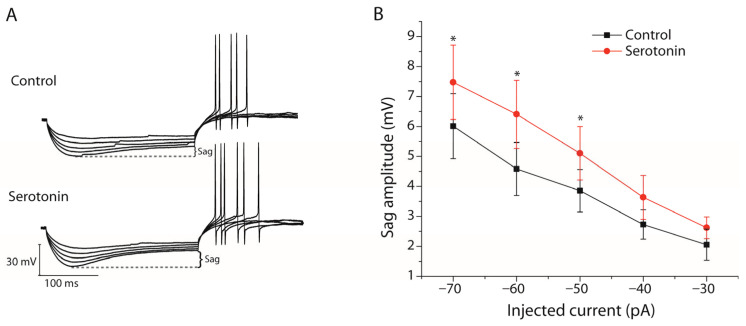
Serotonin enhances voltage sag responses in stellate cells. (**A**) Representative membrane potential responses of a stellate cell to hyperpolarizing current injections under control conditions and after serotonin application (25 µM). Serotonin increased the voltage sag during hyperpolarizing current steps. (**B**) Quantification of sag amplitude at different current injections (−70 to −30 pA) under control conditions and in the presence of serotonin. Sag amplitude was significantly increased at stronger hyperpolarizing steps. Data are presented as mean ± SEM (*n* = 13 cells). * *p* < 0.05 vs. control.

Voltage sag responses were analyzed to further examine whether serotonin modulates HCN channel-mediated Ih currents in stellate cells. Hyperpolarizing current injections evoked a characteristic voltage sag during control recordings, indicative of Ih activation (Figure 7A). Application of serotonin significantly increased sag amplitude at stronger hyperpolarizing current steps. At −70 pA, sag amplitude increased from 6.01 ± 1.08 mV under control conditions to 7.48 ± 1.24 mV in the presence of serotonin (*n* = 13, paired Student’s *t*-test, *p* = 0.0327, Figure 7B). Similarly, at −60 pA, sag amplitude increased from 4.58 ± 0.88 mV to 6.41 ± 1.14 mV (*n* = 13, paired Student’s *t*-test, *p* = 0.0184, Figure 7B), and at −50 pA, from 3.86 ± 0.71 mV to 5.11 ± 0.73 mV (*n* = 13, paired Student’s *t*-test, *p* = 0.05, Figure 7B). Although sag amplitude also tended to increase at −40 pA and −30 pA, these differences did not reach statistical significance. These findings indicate that serotonin enhances voltage sag responses in stellate cells, suggesting an increase in HCN channel-dependent Ih activity.

### 2.5. Serotonin Enhances a Cs^+^-Sensitive Ih Current in Stellate Cells

To investigate how serotonin modulates Ih, activation kinetics were measured before and after serotonin. An appropriate voltage protocol was applied to induce Ih. Voltage steps ranging from −60 mV to −130 mV (5 mV increments) were applied from a −60 mV holding potential. The steady-state activation curve was plotted by measuring the currents at each voltage step.

After electrophysiologically characterizing stellate cells under current clamp in normal aCSF, voltage-clamp experiments were performed. To study Ih in isolation, the aCSF additionally included 1 µM TTX, 1 mM 4-AP, 10 mM TEA to block sodium currents, transient outward current (I_A_), delayed rectifier potassium currents (I_KDR_), respectively. Also were added 5 µM DNQX, 10 µM APV-5 and 1 µM strychnine to block synaptic activities arising from glutamate receptors AMPA and NMDA and glycinergic receptors, respectively. In this way, sodium currents, I_KDR_ and I_A_ currents and synaptic currents were blocked, leaving only leak currents and Ih currents

**Figure 8:**
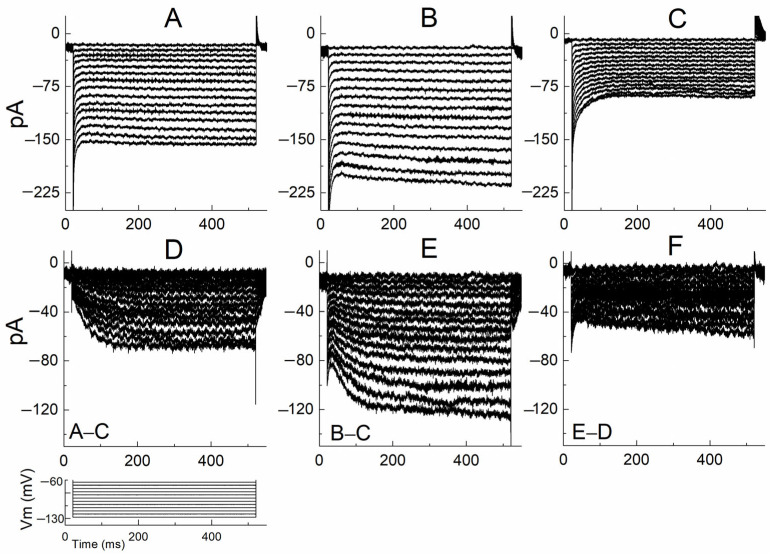
Isolation of serotonin-induced Ih current in stellate cells. (**A**) Representative Ih currents recorded under voltage-clamp conditions in the presence of TTX, 4-AP, TEA, DNQX, APV, and strychnine to block sodium currents, potassium currents (IKDR and IA), and synaptic activity. The voltage protocol used to activate Ih is shown at the bottom. (**B**) Ih currents recorded after application of serotonin (25 µM), showing an increase in the amplitude of the hyperpolarization-activated inward current. (**C**) Currents recorded after application of Cs^+^ (5 mM) to block HCN channels; the remaining currents predominantly represent leak conductance. (**D**) Basal Ih currents obtained by subtracting the Cs^+^-insensitive leak currents (**C**) from the control recordings (**A**). (**E**) Serotonin-modulated Ih currents obtained by subtracting the leak currents (**C**) from the serotonin recordings (**B**). (**F**) Difference currents obtained by subtracting the basal Ih (**D**) from the serotonin-modulated Ih (**E**), representing the additional Ih component induced by serotonin.

Representative current traces recorded before and after serotonin application are shown in Figure 8A and Figure 8B, respectively. Both recordings include Ih and leak currents. When Cs^+^ (5 mM) was applied to block Ih currents, the remaining currents predominantly reflected leak conductance (Figure 8C). Pure Ih currents under control conditions were obtained by subtracting the leak currents (Figure 8C) from the control traces (Figure 8A), as shown in Figure 8D. Similarly, serotonin-modulated Ih currents were isolated by subtracting Figure 8C from Figure 8B (Figure 8E). The difference between the serotonin-modulated Ih (Figure 8E) and control Ih (Figure 8D) represents the additional Ih component induced by serotonin (Figure 8F). These results demonstrate that serotonin enhances a Cs^+^-sensitive Ih current in stellate cells.

**Figure 9:**
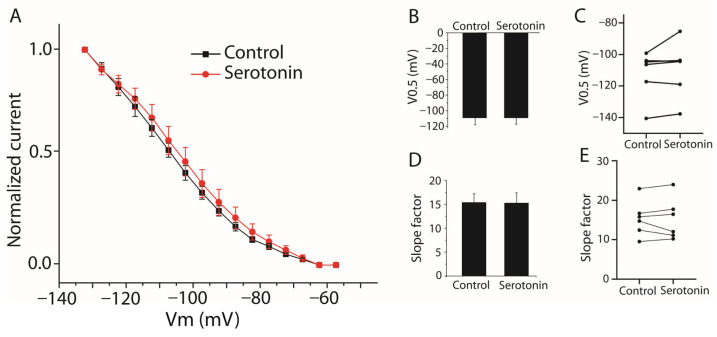
Effect of serotonin on the steady-state activation properties of Ih currents in stellate cells. (**A**) Representative Ih currents evoked by hyperpolarizing voltage steps under control conditions and after serotonin (25 µM) application. The steady-state activation curve was constructed from normalized current amplitudes measured at each voltage step. Currents were normalized to the maximal current obtained at −130 mV and plotted as a function of voltage. The data were fitted with a Boltzmann function to determine the V0.5 and slope factor. (**B**) Summary bar graph comparing the V0.5 between control and serotonin conditions. (**C**) Paired dot plot showing V0.5 values for individual stellate cells (*n* = 6) before and after serotonin application. (**D**) Summary bar graph comparing the slope factor between control and serotonin conditions. (**E**) Paired dot plot showing slope factor values for individual stellate cells (*n* = 6) before and after serotonin application. Serotonin induced a modest depolarizing shift in V0.5, but this difference did not reach statistical significance, and the slope factor was not significantly altered (*n* = 6, paired Student’s *t*-test, *p* > 0.05). Data are presented as mean ± SEM.

Serotonin increased Ih current amplitude (Figure 8). To further characterize the mechanism underlying this increase, we examined the steady-state activation properties of Ih channels. The steady-state activation curve was constructed using normalized current amplitudes measured at each voltage step. Currents were normalized to the maximum current obtained at −130 mV and plotted as a function of voltage. The data were fitted with a Boltzmann function to determine the half-activation voltage (V0.5) and slope factor (k) (Figure 9A). V0.5 was −111.98 ± 6.21 mV before serotonin and −109.06 ± 7.19 mV after serotonin application Although a depolarizing shift of approximately 2.9 mV was observed, this difference did not reach statistical significance (*n* = 6, paired Student’s *t*-test, *p* > 0.05 Figure 9B,C). The slope factor was not significantly altered (control: 15.36 ± 1.85 mV; serotonin: 15.26 ± 2.13 mV; *n* = 6, paired Student’s *t*-test, *p* > 0.05, Figure 9D,E). This shift suggests a modest increase in the fraction of HCN channels available for activation and is consistent with an enhanced depolarizing contribution of Ih during serotonergic modulation.

**Figure 10:**
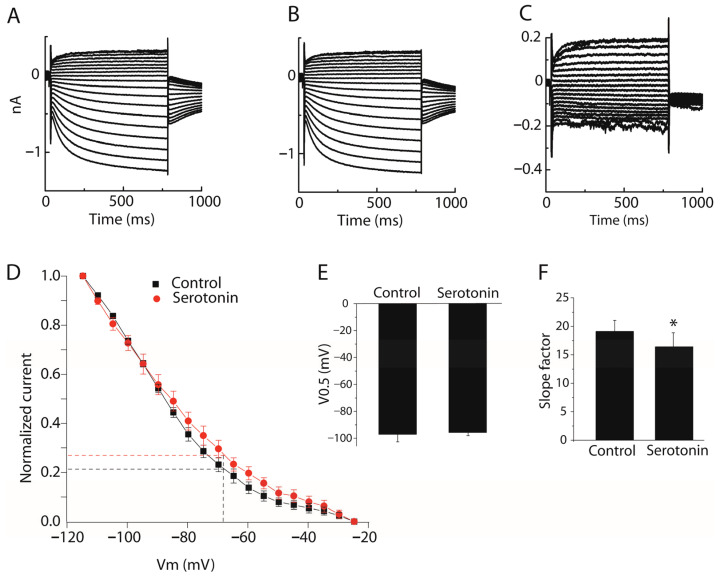
Effect of serotonin on Ih channel availability at near-resting membrane potential in stellate cells. (**A**) Representative Ih current recorded before serotonin application. (**B**) Representative Ih current recorded after serotonin application. (**C**) Serotonin-induced tail currents obtained by subtracting traces in panel A from those in panel B. (**D**) Average steady-state activation (availability) curve (*n* = 6) constructed from normalized tail current amplitudes and fitted with a Boltzmann function. The curve illustrates the fraction of HCN channels available for activation at near-resting membrane potentials under control conditions and after serotonin application. (**E**) Group comparison of the V0.5 between control and serotonin conditions. Serotonin produced a small depolarizing shift in V0.5 that did not reach statistical significance. (**F**) Group comparison of the slope factor between control and serotonin conditions. Serotonin significantly decreased the slope factor, indicating increased voltage sensitivity of Ih activation. Data are presented as mean ± SEM (*n* = 6, paired Student’s *t*-test, * *p* < 0.05 vs. control).

To assess Ih channel availability under near-resting conditions, we also analyzed steady-state activation using a protocol with a holding potential close to the resting membrane potential (−62 mV). Voltage steps ranging from −25 mV to −115 mV in 5 mV increments (750 ms duration) were applied, followed by a step to −77 mV to elicit tail currents. Peak tail current amplitudes were measured at each voltage step and normalized to the maximum tail current value. The normalized values were plotted as a function of voltage and fitted with a Boltzmann function to determine V0.5 and slope factor. Under this protocol, V0.5 values were −97.09 ± 5.62 mV in control and −95.69 ± 1.89 mV after serotonin, with no significant difference (*n* = 6, paired Student’s *t*-test, *p* > 0.05, Figure 10E). However, the slope factor decreased significantly from 19.10 ± 1.95 mV to 16.41 ± 2.31 mV following serotonin application (*n* = 6, paired Student’s *t*-test, *p* < 0.05, Figure 10F), indicating enhanced voltage sensitivity of Ih activation under near-resting conditions. Based on the fitted curves, the predicted fraction of available HCN channels at resting membrane potential increased from approximately 21% to 27% following serotonin application. These findings suggest increased Ih availability at near-resting conditions during serotonergic modulation.

### 2.6. Immunohistochemical Staining

To anatomically support the functional findings described above, immunohistochemical analysis was performed to examine the distribution of 5-HT1A, 5-HT2A, and 5-HT2C receptors in neurons located within the AVCN. Previous studies have shown that calretinin intensely stains auditory nerve fibers and the VCN. In addition, it has been reported that especially octopus neurons in both the AVCN and PVCN regions show a positive immune reaction with calretinin [33,34]. According to these reports, the presynaptic terminals (8th nerve terminals) on the bushy neurons in the AVCN region are positively immunohistochemically stained with calretinin, while their somas are not. In other words, positive staining in the dendrites and presynaptic terminal regions of bushy neurons, but no staining in the soma region, is a distinctive characteristic finding. Based on the above references, neurons whose somas are labeled by calretinin are thought to be stellate cells.

**Figure 11:**
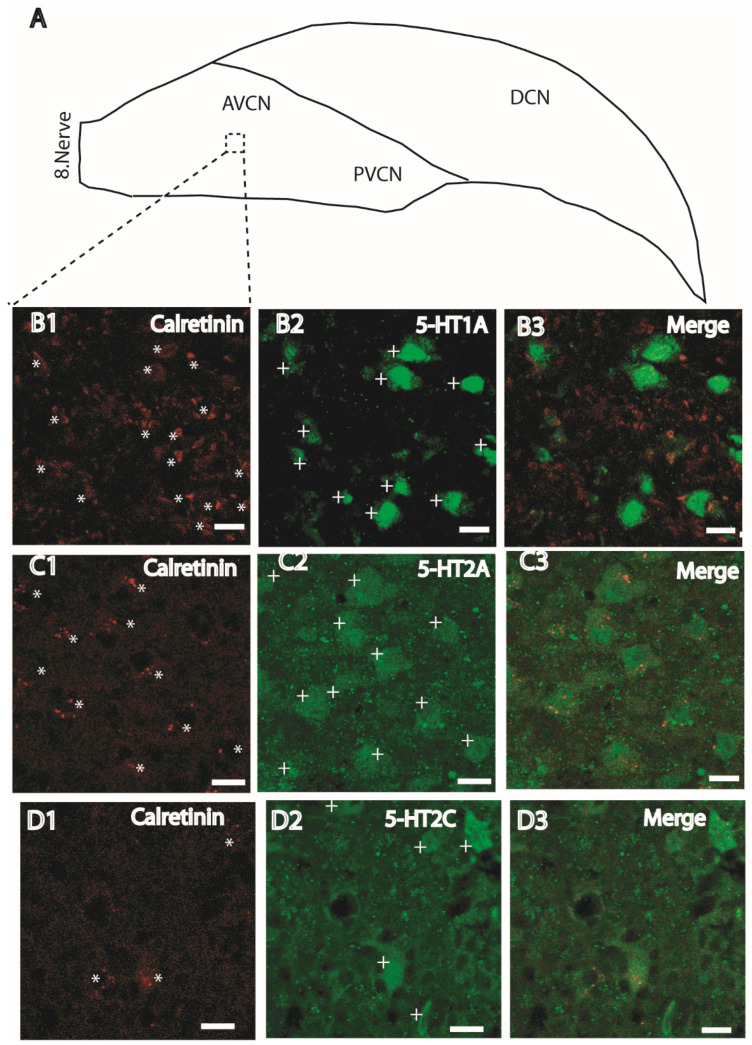
Confocal images of mouse AVCN sections immunolabeled with calretinin, 5-HT1A, 5-HT2A and 5-HT2C. (**A**) Schematic diagram of the cochlear nucleus shows the AVCN region with respect to other CN regions. (**B1**,**C1**,**D1**) Sections were double labelled with calretinin. (**B2**,**C2**,**D2**) 5-HT1A, 5-HT2A, and 5-HT2C staining, respectively. Calretinin labelling was performed to identify the stellate cells in the CN sections. (**B3**,**C3**,**D3**) Merged images showing co-localization of calretinin with 5-HT1A, 5-HT2A, and 5-HT2C receptor labeling, respectively. Asterisks (*) indicate calretinin-positive cell bodies (stellate cells); plus signs (+) indicate cells expressing 5-HT receptor subtypes. Calretinin immunoreactivity is shown in red, and 5-HT receptor labeling is shown in green. Merged images illustrate the overlap of these signals. Scale bar: 20 µm.

The AVCN coronal slices were stained against 5-HT1A, 5HT2A and 5-HT2C receptor subtypes, and each subtype of serotonin receptors was co-stained with calretinin (Figure 11). The cells whose cell bodies, as indicated by asterisks in B1, C1 and D1, are stained with calretinin are thought to be stellate. Cells expressing 5-HT1A, 5-HT2A and 5-HT2C receptors are indicated with plus signs (Figure 11(B2,C2,D2)). The merged images are given in Figure 11(B3,C3,D3). According to the immunohistochemical staining results, it can be suggested that serotonin receptor subtypes (5-HT1A, 5-HT2A and 5-HT2C) are expressed in stellate cells. These findings are consistent with the electrophysiological data.

## 3. Discussion

The present experiments have provided the first direct evidence, using electrophysiological patch-clamp techniques and immunohistochemical staining that serotonin has excitatory effects on stellate cells in the AVCN neurons of mice. Excitatory effects of serotonin on stellate cells appear to occur through 5-HT1A, 5-HT2A and 5-HT2C receptor subtypes. This study demonstrates that serotonin enhances excitability of stellate neurons in the AVCN and is associated with modulation of hyperpolarization-activated cyclic nucleotide-gated (HCN) channels. Serotonin increased Ih current amplitude and produced depolarizing shifts in steady-state activation curves, resulting in increased channel availability at near-resting membrane potentials. Consistent with this mechanism, serotonin also enhanced voltage sag responses during hyperpolarizing current injections, further supporting increased HCN channel activity under current-clamp conditions. These findings support a functional contribution of HCN channels to serotonin-induced excitability in stellate neurons. Together, these findings suggest that Ih contributes to serotonergic modulation within the AVCN.

It has been shown by various molecular and imaging techniques that serotonergic fibers are present in the VCN [12,23,34,35,36]. However, there is limited information on the effects of serotonin on the cells in this region. In a study examining the effects of serotonin on the cochlear nucleus, serotonin was applied iontophoretically and produced both inhibitory and excitatory effects on neurons in this region [15]. In that study, the regions and cell types were not specified, and it was emphasized that the differential effects of serotonin may be related to varying neuronal sensitivities. Furthermore, the researchers noted that serotonin plays a role in modulating cellular signals in hearing-related processes

### 3.1. Effects of Serotonin on Membrane Properties in AVCN Neurons

Previous studies have reported serotonergic modulation in other auditory brainstem nuclei. For example, Felix et al. showed that iontophoretic application of serotonin in dorsal cochlear nucleus (DCN) fusiform cells produced heterogeneous but predominantly excitatory effects [16]. Similarly, whole-cell recordings from principal neurons in the DCN demonstrated that serotonin induced membrane depolarization and increased spontaneous firing frequency [27].

Although these studies focused on DCN neurons, they indicate that serotonergic signaling can enhance neuronal excitability at early stages of auditory processing. The present findings extend this concept to AVCN stellate neurons, demonstrating that serotonin similarly increases excitability in a distinct cochlear nucleus subdivision.

In the current study, serotonin (25 µM) produced a depolarization of approximately 5 mV and was accompanied by a reduction in input resistance. A decrease in input resistance does not necessarily predict reduced firing output, as spike generation depends on the relationship between resting membrane potential and spike threshold, as well as on the recruitment of voltage-dependent conductances. The depolarizing shift induced by serotonin likely positioned stellate cells closer to threshold, thereby facilitating action potential generation despite the moderate reduction in input resistance.

In addition, serotonergic modulation of HCN/Ih channels can alter subthreshold membrane dynamics and influence the probability of reaching threshold during depolarizing inputs. Together, these mechanisms provide a plausible explanation for the observed increase in firing following serotonin application. Consistent with these membrane potential changes, serotonin also increased action potential firing in stellate cells. Quantitative analysis of firing frequency confirmed that serotonergic depolarization translates into enhanced spike output, supporting the interpretation that serotonin increases the excitability of AVCN stellate neurons.

### 3.2. Mechanistic Insight: Direct Action of Serotonin

In current-clamp experiments, bath application of 25 µM serotonin produced a significant depolarization in stellate cells and increased their spontaneous firing frequency. The persistence of serotonin’s depolarizing effects in stellate cells in the presence of TTX, 4-AP, TEA and synaptic receptor antagonists (DNQX, APV, and strychnine) strongly suggests that serotonin’s excitatory effects are mediated directly through postsynaptic serotonin receptors rather than via altered synaptic input.

### 3.3. Receptor-Specific Mechanisms

Molecular studies have previously identified the presence of 5-HT1A, 5-HT2A, and 5-HT2C receptors in the AVCN [30,31,32]; however, their functional electrophysiological contributions in stellate cells had not been directly examined. Our results demonstrate that all three receptor subtypes contribute to serotonin-induced excitation, with 5-HT1A receptors mediating a substantial portion of the depolarizing response. Pharmacological blockade with WAY-100635 significantly attenuated serotonin-induced depolarization and reduced the associated inward current, indicating a clear excitatory contribution of 5-HT1A receptor activation.

Although 5-HT1A receptors are classically described as Gi/o-coupled receptors that reduce adenylyl cyclase activity and intracellular cAMP levels [37], the electrophysiological outcome of GPCR activation cannot be predicted solely from second-messenger coupling. Rather, the membrane response depends critically on the downstream ion channels expressed in the postsynaptic neuron.

In many neuronal populations, 5-HT1A receptor activation produces inhibition via recruitment of G protein-activated inwardly rectifying potassium (GIRK) channels, leading to outward K^+^ currents and hyperpolarization [38,39]. However, this effect is cell-type dependent and not universal. In neurons in which HCN conductance plays a prominent role in subthreshold membrane dynamics, modulation of Ih can produce a net depolarizing effect even when the initiating receptor is Gi/o-coupled [27].

Serotonergic enhancement of Ih has previously been demonstrated in the auditory brainstem. Tang and Trussell (2015) reported that serotonin depolarizes dorsal cochlear nucleus fusiform cells by enhancing HCN-mediated currents and altering activation properties [27]. Our findings in AVCN stellate cells are mechanistically consistent with this framework. We observed a robust serotonin-induced depolarization (~5 mV), a predominantly Cs^+^-sensitive inward current, and an increase in Ih amplitude. These data suggest that, in stellate cells, serotonergic excitation is mediated primarily through modulation of depolarizing conductances rather than through classical GIRK-dependent inhibition. These findings further illustrate that serotonergic receptor classification based on canonical second-messenger coupling does not necessarily predict functional polarity at the membrane level, emphasizing the importance of cell-specific ion channel context.

Thus, the apparent discrepancy between canonical Gi/o coupling and the observed excitation can be reconciled by considering the ion channel repertoire of the target neuron. In stellate cells, where HCN channels substantially contribute to resting membrane potential and subthreshold responsiveness, enhancement of Ih availability can shift membrane potential toward spike threshold and increase firing probability.

The contribution of 5-HT2A and 5-HT2C receptors further supports a multi-receptor mechanism. Both antagonists (4F-4PP oxalate and SB-242084) partially reduced serotonin-induced depolarization and inward current, indicating additive or convergent receptor involvement. As Gq-coupled receptors, 5-HT2A and 5-HT2C activation may modulate ion channel activity through suppression of K^+^ currents or facilitation of non-selective cation conductances [40,41], thereby complementing HCN-dependent effects.

Taken together, these findings demonstrate that multiple serotonin receptor subtypes—5-HT1A, 5-HT2A, and 5-HT2C—cooperatively enhance excitability in AVCN stellate cells. Rather than acting through a single canonical pathway, serotonergic modulation appears to reflect receptor-specific signaling converging on membrane conductances that regulate subthreshold excitability.

### 3.4. Serotonin Modulation of Ih Currents via HCN Channels

The present study demonstrates that serotonin enhances the excitability of stellate cells in the AVCN primarily through modulation of HCN channel-mediated Ih currents. Pharmacological blockade with extracellular Cs^+^ abolished approximately 95% of the serotonin-induced depolarization and inward current, strongly supporting the conclusion that Ih represents the principal ionic substrate underlying serotonergic excitation in these neurons. Voltage-clamp recordings further revealed that serotonin significantly increased Ih current amplitude. This increase in conductance provides an additional depolarizing drive at subthreshold membrane potentials and is sufficient to enhance neuronal excitability.

In addition to the voltage-clamp measurements, voltage sag responses during hyperpolarizing current injections were quantified in current-clamp recordings. Serotonin significantly increased sag amplitude at stronger hyperpolarizing current steps, providing further functional evidence for enhanced HCN channel activity. Voltage sag is a classical electrophysiological signature of Ih activation, and the observed increase in sag amplitude is therefore consistent with our voltage-clamp findings showing increased Ih amplitude and altered activation properties following serotonin application. Together with the increase in Ih amplitude observed under voltage-clamp conditions, the enhancement of voltage sag further supports the interpretation that serotonergic modulation increases HCN channel activity in AVCN stellate cells.

Ih was directly isolated under voltage-clamp conditions and pharmacologically verified via Cs^+^ sensitivity. Although Cs^+^ is not entirely specific for HCN channels, it is widely used as a classical Ih blocker at millimolar concentrations. Future studies employing complementary pharmacological tools, such as ZD7288 or subtype-specific approaches, may further refine the contribution of individual HCN channel isoforms to serotonergic modulation.

Analysis of steady-state activation under a standard protocol revealed a modest depolarizing trend in V0.5; however, this shift did not reach statistical significance, and the slope factor remained unchanged. These findings suggest that serotonin does not produce a large displacement of the activation midpoint under these conditions.

Importantly, under near-resting membrane potential conditions, serotonin significantly reduced the slope factor of the activation curve without significantly altering V0.5. This reduction in slope reflects increased voltage sensitivity of Ih gating and may amplify the depolarizing influence of Ih within a narrow physiological voltage range. Consequently, serotonergic modulation appears to primarily fine-tune channel gating dynamics rather than shift the activation threshold itself, thereby enhancing subthreshold membrane responsiveness even in the absence of large V0.5 shifts.

Together, these results indicate that serotonergic excitation of stellate cells is mediated predominantly by increased Ih amplitude and altered activation kinetics under near-resting conditions. Rather than producing a robust shift in activation threshold, serotonin appears to fine-tune HCN channel behavior to enhance depolarizing influence within a physiologically relevant voltage range. These findings are consistent with previous reports demonstrating serotonergic enhancement of Ih in other auditory nuclei. Tang and Trussell (2015) showed that serotonin increases Ih in fusiform cells of the dorsal cochlear nucleus, while Bal and Oertel (2000) emphasized the importance of HCN-mediated currents in auditory temporal precision [27,28]. Extending these observations, the present study identifies HCN channel modulation as a key contributor to serotonergic regulation of stellate cell excitability in the AVCN.

### 3.5. Immunohistochemical Analysis

Immunohistochemical analysis revealed the presence of 5-HT1A, 5-HT2A, and 5-HT2C receptor immunoreactivity in neurons located within the AVCN. Calretinin labeling was used to aid in the identification of stellate cells, based on previously described staining patterns in the cochlear nucleus. Although calretinin labeling intensity appeared to vary between panels, this variability most likely reflects differences in section depth, local neuronal density, and confocal acquisition settings rather than biological inconsistency. All images within each staining set were acquired using comparable imaging parameters.

The immunoreactivity for 5-HT1A in AVCN neurons observed in our study is consistent with previous findings in Galago (bush baby), cat, guinea pig, and rat [30,32,42,43]. Meanwhile, the moderate immunoreactivity observed for 5-HT2A aligns with prior studies in rats [31,32]. To our knowledge, there are no previous reports describing 5-HT2C receptor expression specifically in AVCN neurons, and our findings therefore provide initial anatomical evidence for its presence in this region.

Serotonin receptor immunoreactivity was observed in somatic and proximal dendritic regions. Although serotonin receptors are classically described as membrane proteins, G protein-coupled receptors are known to undergo ligand-dependent internalization and intracellular trafficking, which may result in cytoplasmic and vesicular localization patterns. Thus, somatic cytoplasmic labeling does not exclude membrane expression but may reflect receptor trafficking dynamics or intracellular receptor pools.

The primary antibodies used in this study were selected based on manufacturer validation data and prior peer-reviewed publications reporting comparable labeling patterns. While immunohistochemistry does not establish functional receptor activity, the anatomical distribution observed here is consistent with the electrophysiological data demonstrating functional contributions of these receptor subtypes in stellate cells.

Taken together, these findings support the presence of 5-HT1A, 5-HT2A, and 5-HT2C receptors in AVCN neurons and are compatible with the functional modulation observed in patch-clamp recordings.

We did not perform triple co-localization analysis within the same neuron, and therefore cannot directly determine the proportion of cells co-expressing all three receptor subtypes. The primary aim of the immunohistochemical experiments was to establish the anatomical presence of each receptor subtype within AVCN neurons. Functional contributions of these receptors were independently evaluated through selective pharmacological antagonists in electrophysiological recordings. Future studies employing quantitative co-localization approaches would further clarify the extent of receptor co-expression at the single-cell level.

### 3.6. Functional Significance of Serotonergic Modulation

In the present study, serotonin increased the excitability of stellate cells in the AVCN through the activation of multiple 5-HT receptor subtypes and modulation of HCN channel function. This finding is consistent with previous in vivo and in vitro studies demonstrating that serotonin enhances excitability in neurons of the dorsal cochlear nucleus (DCN) [16,27], supporting the broader role of serotonergic signaling in early auditory processing.

Stellate cells play an important role in encoding amplitude and temporal aspects of acoustic signals and in relaying this information to higher-order auditory centers. By increasing subthreshold depolarizing drive through Ih enhancement and altered activation kinetics, serotonin may dynamically regulate the responsiveness of stellate cells to incoming auditory inputs. Such modulation suggests that serotonergic tone can fine-tune neuronal excitability within physiologically relevant voltage ranges rather than simply shifting activation thresholds.

These findings contribute to the growing body of evidence that neuromodulatory systems shape auditory processing at early brainstem levels. Serotonin appears to act as a context-dependent regulator of neuronal gain in the VCN, potentially adjusting the balance between spontaneous activity and stimulus-driven responses.

Alterations in serotonergic signaling have been implicated in auditory pathologies, including tinnitus and hyperacusis [17,19,44]. Although the present study does not directly model tinnitus, increased excitability of auditory brainstem neurons has been proposed as a contributing mechanism in tinnitus development. Previous reports have suggested that serotonin-induced depolarization of DCN fusiform cells may participate in maladaptive hyperexcitability [27,45]. Similarly, enhanced serotonergic activity in the VCN has been observed in acoustic trauma and salicylate-induced tinnitus models [19,20,21]. The present findings raise the possibility that serotonergic modulation of stellate cell excitability may represent one component of the broader neural plasticity underlying tinnitus-related hyperexcitability.

## 4. Materials and Methods

All procedures were approved by the local animal use committee of University of Gaziantep (Gaziantep, Turkey) (Protocol date/no: 01.10.2019/112). Experiments were performed in strict accordance with the guidelines therein. Albino BALB/c mice at the ages of 12–17 days were used in this study. Animals were decapitated under xylazine HCl (5 mg/kg) and ketamine (60 mg/kg) anesthesia and then the head was immersed in continuously oxygenated (95% O_2_/5% CO_2_) normal artificial cerebrospinal fluid (aCSF). After the skull was removed, the brainstem was cut coronally between the superior colliculus and inferior colliculus (mid-collicular level) with an approximately 60° angle. The brainstem was glued onto the Teflon block using cyanoacrylate glue (SUPERGLUE), with the inferior colliculus facing down. This Teflon block was placed into the bath of a vibratome (Frederick Haer, New Brunswick, ME, USA), which was filled with continuously oxygenated normal aCSF, and 175 μm-thick coronal slices were obtained. Then, the slices were incubated for approximately 30 min in the continuously oxygenated normal aCSF at room temperature and were then transferred into a recording chamber with a volume of 0.3 mL solution. The temperature of the perfused aCSF was kept constant at 33 °C with a temperature controller. AVCN neurons were viewed under an AxioScope FS upright microscope (Carl Zeiss, Jena, Germany) equipped with differential interference contrast optics and a 63× water-immersion lens. There are three cell types with different biophysical properties in the ventral cochlear nucleus, which are stellate, bushy and octopus cells [2,7,31]. Stellate cells are found together with bushy cells in the AVCN region. Electrophysiological properties were used to distinguish stellate and bushy cells. Electrophysiological recordings showed that bushy cells fire only one or two action potentials in response to square current pulses, whereas stellate cells fire tonically in response to a suprathreshold depolarizing current pulse [4,5,46].

### 4.1. Solutions and Chemicals

The pipette solution contained (in mM): 108 potassium gluconate, 9 HEPES, 4.5 MgCl_2_, 9 EGTA, 14 phosphocreatine (tris salt), 4 ATP (Na salt), and 0.3 GTP (tris salt). The pH of the solution was adjusted to 7.35–7.40 using KOH [28]. ATP, GTP, and phosphocreatine were added to the pipette solution so that stable recordings could be obtained for long times [47]. The pipette solution was prepared as a stock solution and kept at −20 °C.

As a perfusion solution in the current-clamp experiments, aCSF was used. The normal aCSF solution is composed of (in mM) 138 NaCl (Merck, Darmstadt, Germany), 4.2 KCl (Merck), 2.4 CaCl_2_ (Fluka), 1.3 MgSO_4_ (Merck), 10 HEPES (Merck), 10 glucose (Sigma Aldrich, Burlington, MA, USA), saturated with 95% O_2_/5% CO_2_. The pH of the solution was adjusted to be 7.40 with NaOH [7,27].

In order to study Ih in isolation aCSF additionally included 1 µM tetrodotoxin (TTX) (Alexis Biochemicals (USA) to block sodium currents), 1 mM 4-aminopyridine (4-AP) (Sigma-Aldrich) to block transient outward current (I_A_), 10 mM tetraethyl ammonium (TEA) (Fluka) to block delayed rectifier potassium currents (I_KDR_), 5 µM 6,7-Dinitroquinoxaline-2,3-dione (DNQX), 10 µM 2-amino-5-phosphonopentanoic acid (APV-5) and 1 µM strychnine to block synaptic activities arising from glutamate receptors AMPA and NMDA and glycinergic receptors, respectively. 5 mM cesium chloride (CsCl) (Sigma-Aldrich) was added to block Ih. The osmolarity of both solutions was kept between 295 and 310 mOsm/kg. Both solutions were prepared freshly every day and were oxygenated 30 min before the experiment.

The concentration of 25 µM serotonin was selected based on previous electrophysiological studies in acute brain slice preparations demonstrating reliable receptor-mediated responses under similar recording conditions [48]. In slice experiments, diffusion barriers and uptake mechanisms often necessitate micromolar concentrations to achieve stable and reproducible effects.

### 4.2. Patch Clamp Recordings

Recordings were performed in P12–P17 mice, a developmental window following hearing onset in which intrinsic neuronal properties are largely established while slice preparation remains technically optimal. The patch electrodes pulled from borosilicate glass with tip resistances of 4–7 MΩ after filling with the pipette solution were used in the experiments. In normal extracellular saline, this solution yielded a junction potential of −12 mV that was taken into account. Whole cell current- and voltage-clamp recordings were performed with an Axopatch 200B amplifier controlled by a PC through a Digidata 1440 computer interface and pClamp 10.7 software (Axon Instruments, Foster City, CA, USA). Before going to the whole-cell configuration, high-resistance seals (gigaseals) (>1 GΩ) were obtained. Series resistance and capacitance compensation were applied on-line.

A series of 500 ms current steps (starting at −70 pA and incrementing by 20 pA) was injected into stellate cells to assess firing responses. Input resistance (Rin) was calculated from voltage responses to hyperpolarizing current steps. For each recording, the steady-state membrane potential change (ΔV) evoked by a given current injection (ΔI) was measured, and Rin was computed according to Ohm’s law (Rin = ΔV/ΔI). When multiple hyperpolarizing steps were available, Rin values were calculated for each step and averaged to obtain a single Rin value per cell. To minimize contamination by voltage-dependent conductances, Rin was estimated using small hyperpolarizing steps within the linear range of the I–V relationship. This approach is equivalent to estimating the slope of the linear portion of the I–V relationship around resting potentials. We note that Rin was estimated from steady-state voltage deflections at hyperpolarizing steps (linear range) and may not fully capture dynamic, voltage-dependent conductances that shape excitability near threshold.

### 4.3. Immunohistochemistry

Immunohistochemistry experiments were performed similarly to our previous study [49]. 30–35 day old mice were decapitated after general anesthesia induced by intramuscular injection of a combination of xylazine HCl (5 mg/kg) and ketamine (60 mg/kg). Brainstems containing CNs were dissected and immediately fixed in ice-cold 4% paraformaldehyde solution prepared in 0.1 M phosphate buffer (PB; pH: 7.4) for 30 min. The tissues were transferred to ice-cold 30% sucrose in 0.1 M phosphate buffer with 0.1% Na-azide for cryoprotection overnight. The brainstems with CN were cut on a freezing microtome with the temperature adjusted to −20 °C into a series of adjacent 20 μm-thick coronal sections and were then thaw-mounted on polylysine-coated glass slides. Sections were incubated for at least 2 h in a blocking solution that contained 0.1% Triton X, 0.1% sodium azide, 3% bovine serum albumin, and 5% normal goat serum in PBS. Tissues were incubated overnight at +4 °C in the solutions with the following primary antibodies. The CN sections were double-stained with serotonin receptor antibodies (St John’s Laboratory, London, UK) and calretinin antibody (Santa Cruz Biotechnology, Dallas, TX, USA). After rinsing with PBS, the sections were incubated for 2 h at room temperature in PBS including either 3% goat serum, 3% bovine serum albumin, 0.1% Tween-20 and 0.1% sodium azide with the following secondary antibodies (Alexa Fluor, Invitrogen, Carlsbad, CA, USA). Details of the primary and secondary antibodies are provided in Table 1. The sections were then rinsed with PBS. Images were captured using a scanning confocal microscope (Zeiss LSM 900, Zeiss, Oberkochen, Germany). We recorded both single optical sections and image stacks (z-step 0.14 m).

Statistical analyses were performed using IBM SPSS Statistics (Version 23.0; IBM Corp., Armonk, NY, USA). Data are presented as mean ± standard error of the mean (SEM), with ‘n’ representing the number of recorded cells. For paired comparisons (e.g., measurements obtained from the same cell before and after drug application), differences were evaluated using a paired Student’s *t*-test. A *p* < 0.05 was considered statistically significant.

## 5. Conclusions

This study demonstrates that serotonin enhances the excitability of stellate cells in the mouse AVCN through activation of 5-HT1A, 5-HT2A, and 5-HT2C receptors. Electrophysiological recordings revealed that serotonin induces membrane depolarization, increases firing probability, and reduces input resistance. Pharmacological and voltage-clamp analyses indicate that this excitatory effect is largely mediated by modulation of HCN channel-dependent Ih currents.

Serotonin increased Ih amplitude and altered activation kinetics under near-resting membrane potential conditions, supporting the interpretation that serotonergic modulation enhances subthreshold depolarizing drive within a physiologically relevant voltage range rather than producing a large shift in activation threshold.

Together, these findings identify multi-receptor serotonergic control of HCN-dependent excitability as a key mechanism regulating stellate cell responsiveness in the AVCN.

Several limitations of the present study should be acknowledged. Combined receptor blockade experiments and pharmacological inhibition of HCN channels using selective blockers such as ZD7288 were not performed. Therefore, future studies will be required to further clarify the relative contribution of different serotonin receptor subtypes and the precise role of HCN channels in serotonergic modulation of stellate cell excitability.

## Figures and Tables

**Table 1:** List of primary and secondary antibodies used in immunohistochemical studies.

Antibodies		Dilution
5-HT1A (Primary)	Anti-rabbit 5-HT1A receptor antibody	1/200
(Secondary)	Goat anti-rabbit IgG Alexa Fluor^®^ 488	1/100
5-HT2A (Primary)	Goat anti-rabbit-5-HT2A Antibody	1/200
(Secondary)	Goat anti-rabbit IgG Alexa Fluor^®^ 488	1/100
5-HT2C (Primary)	Goat anti-rabbit-5-HT2C Antibody	1/100
(Secondary)	Goat anti-rabbit IgG Alexa Fluor^®^ 488	1/100
Calretinin (Primary + Secondary)	Anti-mouse IgG Alexa Fluor^®^ 594	1/50

## Data Availability

The data supporting the findings of this study are available from the corresponding author upon reasonable request, due to the large size of electrophysiological recording files.

## Abbreviations

The following abbreviations are used in this manuscript:

CN	Cochlear nucleus
VCN	Ventral cochlear nucleus
DCN	Dorsal cochlear nucleus
AVCN	Anteroventral cochlear nucleus
PVCN	Posteroventral cochlear nucleus
5-HT	5-Hydroxytryptamine (serotonin)
5-HT1A	5-Hydroxytryptamine receptor 1A
5-HT2A	5-Hydroxytryptamine receptor 2A
5-HT2C	5-Hydroxytryptamine receptor 2C
HCN	Hyperpolarization-activated cyclic nucleotide–gated channel
Ih	Hyperpolarization-activated current
TTX	Tetradotoxin
4-AP	4-aminopyridine
aCSF	Artificial cerebrospinal fluid
DNQX	Dinitroquinoxaline-2,3-dione
TEA	Tetraethyl ammonium
APV-5	2-amino-5-phosphonopentanoic acid
I_KDR_	Delayed rectifier potassium currents
I_A_	Transient outward current
pA	Picoampere
mV	Millivolt
Vm	Membrane potential
